# Elevated lipoprotein(a) as a new risk factor of cerebral venous sinus thrombosis: association with fibrin clot properties

**DOI:** 10.1007/s11239-018-1769-0

**Published:** 2018-12-03

**Authors:** Anna Aleksandra Skuza, Maciej Polak, Anetta Undas

**Affiliations:** 1 Institute of Cardiology, Jagiellonian University Medical College, 80 Pradnicka St, 31-202 Cracow, Poland; 2 Department of Epidemiology and Population Studies, Jagiellonian University Medical College, Krakow, Poland; 3 John Paul II Hospital, Krakow, Poland

**Keywords:** Cerebral venous sinus thrombosis, Lipoprotein(a), Recurrence, Fibrin clot

## Abstract

Elevated lipoprotein(a) [Lp(a)] has been reported to be associated with prothrombotic clot phenotype. We hypothesized that increased Lp(a) contributes to cerebral venous sinus thrombosis (CVST) and its recurrence in relation to clot features. In 80 consecutive patients (aged 39.36 ± 10.18 years, 61 women) following the first CVST after anticoagulation withdrawal, we assessed Lp(a) levels and plasma clot properties. Recurrence of CVST was recorded during follow-up (median 53, interquartile range 40–59 months). Lp(a) levels were inversely associated with clot permeability (K_s_, r = − 0.58, P < 0.001) and the rate of D-dimer release from clots in the presence of tissue plasminogen activator (r = − 0.27, P = 0.017) along with increased maximum absorbance of fibrin gels (r = 0.42, P < 0.001) and maximum D-dimer levels achieved during lysis (D–D_max_, r = 0.29, P = 0.01). Recurrence of CVST was observed in 12 patients (15%) after median follow-up of 26 months. Lp(a) concentrations were higher in patients with recurrence of CVST compared to the remainder (14.15 [8.85–25.25] vs. 28.3 [18.9–35.6] mg/dL; P = 0.001). The risk of recurrent CVST was fourfold higher among 17 (21.25%) patients with Lp(a) > 30 mg/dL compared to the remainder (adjusted hazard ratio, 3.9; 95% confidence interval [CI] 1.23–12.4). Recurrence of CVST was associated with 14% lower K_s_ (P = 0.001) and 10% higher D–D_max_ (P = 0.008), with no differences in other clot features. Multiple logistic regression model showed that CVST recurrence was independently associated with Lp(a) (odds ratio 1.09, 95% CI 1.02–1.16). Increased Lp(a) characterizes subjects at elevated risk of recurrent CVST after anticoagulation withdrawal, which could be partly explained by formation of denser fibrin clots.

## Highlights

- In patients with cerebral venous sinus thrombosis (CVST) elevated lipoprotein(a) is associated with unfavorable fibrin clot features.
- Patients with lipoprotein(a) > 30 mg/dL have a 3.9-fold higher risk of recurrent CVST.
- It might be speculated that CVST patients with lipoprotein(a) > 30 mg/dl should be anticoagulated on the long-term basis.

## Introduction

Cerebral venous sinus thrombosis (CVST) is a rare thrombotic disease with its estimated annual incidence of 3–4 cases per million [1]. However, recent studies suggested much higher incidence from 13.2–15.7 cases per million [2, 3]. The clinical presentation depends on the site and extension of the affected sinuses in CVST as well as on the presence of venous collaterals. It varies considerably from the most common severe headache to focal neurological deficits [4]. Several risk factors for CVST have been recognized including oral contraceptive use, pregnancy, infections, inflammatory diseases, and thrombophilia [1, 5]. The overall incidence of recurrent venous thromboembolism in patients with CVST was about 2.03 per 100 person-years and it was highest in the first year after discontinuation of anticoagulant therapy [6].

In 2015 a novel risk factor for CVST unrelated to cancer and trauma was identified, the so-called prothrombotic fibrin clot phenotype [7]. It has been found that faster formation of denser plasma fibrin clots displaying reduced susceptibility to lysis characterizes patients with CVST, and importantly, denser clot structure may predispose to recurrence of CVST. Similar clot features have been reported in patients with cardiovascular disease, including ischemic stroke, and importantly, a predictive value of increased clot density and impaired lysability has been shown in patients with venous thromboembolism (VTE) [8–10]. One of several factors unfavorably affecting plasma clot properties is elevated lipoprotein(a) [Lp(a)] that consists of a low-density lipoprotein (LDL) and apolipoprotein(a) [apo(a)] bound to an apolipoprotein B-100 by a disulfide linkage [11]. The apo(a) protein contains a varying number of kringle domains, which are homologous with kringle domains IV and V in the plasminogen particle [12, 13]. The levels of Lp(a) are highly heritable, exceeding 90% in European populations [14, 15]. The similar structure of apo(a) and plasminogen suggests that Lp(a) may inhibit fibrinolysis [16, 17]. However, on a molar basis, plasminogen is almost always in excess of apo(a), which calls into question its in vivo potency in inhibiting plasminogen activity [18].

Evidence linking increased Lp(a) with venous thrombosis is inconsistent [19–21]. Associations of elevated Lp(a) with CVST are unclear. To our knowledge, there have been case reports on the occurrence of CVST in patients with markedly increased Lp(a) levels [22–24] with one case of recurrence of CVST [23].

Elevated Lp(a) levels have been reported to be associated with decreased clot permeability and susceptibility to lysis in apparently healthy individuals, patients with advanced coronary artery disease and acute ischemic stroke [25–27] as well as those with residual vein obstruction [28]. Given associations of Lp(a) with prothrombotic clot phenotype and its presence in patients following CVST, we hypothesized that elevated Lp(a) can contribute to CVST and its recurrence in association with unfavorable fibrin clot features. Therefore, the aim of the current study is to investigate the links of Lp(a) with CVST in a cohort study.

## Methods

Between January 2006 and May 2017, 80 consecutive adult patients with the first-ever CVST episode were recruited at the Center for Coagulation Disorders in Krakow, including a group reported in 2015 [7]. We enrolled additional 30 patients and collected data for long-term follow-up. CVST was objectively documented by computed tomography angiography, magnetic resonance imaging or magnetic resonance angiography. Exclusion criteria were age above 60 years, current anticoagulation, recent trauma, other thromboembolic events such as deep vein thrombosis, pulmonary embolism and myocardial infarction in the past, severe inflammatory diseases and known malignancy. Patients were eligible after at least 3 months of anticoagulant therapy and at least 4 weeks since the last dose of an anticoagulant, mostly vitamin K antagonists. Demographic data, location of thrombosis and medical history focusing on potential risk factors for CVST were recorded.

Follow-up started after withdrawal of anticoagulant therapy and was recorded every 6 months by visit to our center or by telephone contact. Documented recurrences of symptomatic CVST by imaging studies were recorded. Follow-up was censored at the time of recurrence.

The bioethical committee approved the study and its participants gave informed consent.

### Laboratory investigations

Fasting blood sample were drawn in the morning. Full blood cell count, creatinine, glucose, international normalized ratio (INR) and activated partial thromboplastin time (APTT) were assessed by standard laboratory assays. Fibrinogen was determined by the Clauss method. High-sensitivity CRP was evaluated by nephelometry. Plasma D-dimer, plasminogen activator inhibitor-1 antigen (PAI-1:Ag), tissue-type plasminogen activator antigen (tPA:Ag) were determined using commercially available immunoenzymatic assays. Lipoprotein(a) was measured by an immunoenzymatic assay (DRG Diagnostics, Marburg, Germany). Thrombophilia screening, including protein C, protein S, and antithrombin deficiencies, antiphospholipid antibodies, factor (F)V Leiden, and prothrombin 20210A polymorphisms were performed in all study participants as described previously [9, 29].

To evaluate fibrin clot properties, blood samples (vol/vol, 9:1 of 3.2% trisodium citrate) were centrifuged at 2560 g for 20 min and the supernatant was aliquoted and stored at − 80 °C. All fibrin variables were assessed in duplicate. Interassay variability for all fibrin variables was < 8%.

### Clot permeation

Briefly, to 120 µL citrated plasma 20 mmol/L calcium chloride and 1 U/mL human thrombin were added. After incubation, the plasma clot was percolated with Tris buffer and its volume flowing through the gels were measured. A permeation coefficient (K_s_), was calculated from the equation: K_s_ = Q × L × η/t × A × ∆p, where Q is the flow rate in time t; L is the length of a fibrin gel; η is the viscosity of liquid (in poise); A is the cross-sectional area (in cm^2^) and ∆p is differential pressure (in dyne/cm^2^). The lower K_s_, the more tightly packed a fibrin structure.

### Turbidity measurements

Briefly, plasma citrated samples were mixed 2:1 with Tris buffer, which contained 0.6 U/mL human thrombin and 50 mM calcium chloride. Absorbance was read at 405 nm. The lag phase of the turbidity curve, and the maximum absorbance of a gel (∆Abs) at the plateau phase were recorded.

### Plasma clot lysis assays

To assess the efficiency of clot lysis two methods were used. Briefly, clot lysis time (CLT) was measured in the assay in which citrated plasma was mixed with 15 mmol/L calcium chloride, 0.6 pmol/L human tissue factor ,12 µmol/L phospholipid vesicles and 60 ng/mL recombinant tPA (rt-PA).The turbidity was measured at 405 nm at 37 °C. CLT was defined as the time from midpoint of the clear-to-maximum turbid transition to the midpoint of maximum-turbid-to-clear transition. In the second assay to measure fibrinolysis after clot formation and stabilization, fibrin clots, formed as for permeability evaluation, were perfused with a Tris buffer containing 0.2 µmol/L rt-PA D-dimer concentrations were measured every 20 min. The measurement was stopped when the gel collapsed under the pressure. Maximum rate of increase in D-dimer levels (D–D_rate_) and maximum D-dimer levels (D–D_max_) were analyzed.

### Statistical analysis

Continuous variables were presented as means (standard deviation) or median (interquartile range), as appropriate. The Shapiro–Wilk test was used to test the normal distribution of variable. Categorical variables were reported as percentage. The Chi square test or Fisher exact test were used to compare the distribution of categorical variable. Analysis of variance for continuous variables was used to assess intergroup differences. Analysis of covariance was used to compare the mean difference in fibrin clots properties between groups after adjustment for fibrinogen. Correlations were assessed by the Pearson correlation coefficient. Multiple logistic regression analysis was performed to determine the effect of Lp(a) and fibrin clot properties on the rate of recurrences. Each variable was assessed by model with fibrinogen and sex and those < 0.05 were entered into multivariable analysis. The discrimination ability of a logistic model was used to report the area under the ROC curve with 95% CI. Statistical analyses were performed using SPSS 23.0 (SPSS Inc., Chicago, IL, USA). P-values < 0.05 were accepted as statistically significant.

## Results

Eighty patients aged 39.36 ± 10.18 years following the first documented episode of CVST were studied (Table 1). Most patients were women (78.75%), including 25 (39.68%) on oral contraceptives and 14 (22.22%) diagnosed while pregnant. A positive family history of VTE was noted in 19 subjects (23.75%). The CVST occurred during infection in 8 (10%) patients. Concomitant occurrence of VTE was observed in 9 (11.25%) patients. 12 (15%) patients were carriers of factor V Leiden, 5 (6.25%) patients had 2021A prothrombin mutation, 5 (6.25%) had deficiency of antithrombin, protein C or S, while antiphospholipid syndrome (APS) was observed in 5 (6.25%) patients.

**Table 1:** Characteristics of patients with CVST with or without recurrence during follow-up

	Patients with CVST (n = 80)	Without recurrence (n = 68)	With recurrence (n = 12)	P*
Age, years	39.36 ± 10.18	39.49 ± 10.16	38.67 ± 10.71	0.8
Female, n(%)	61 (76.25)	55 (88.8)	6 (50)	0.02
Body mass index, kg/m^2^	27.39 ± 4.33	27.24 ± 4.25	28.25 ± 4.85	0.46
Risk factors of CVST, n(%)				
Oral contraceptive	25 (31.25)	23 (33.82)	2 (16.67)	0.24
Past pregnancy	14 (17.5)	11 (16.18)	3 (25)	0.46
Cigarette smoking	18 (22.5)	16 (23.53)	2 (16.67)	0.6
Family history of thrombosis	18 (22.5)	14 (20.6)	4 (33.33)	0.33
Thrombophilia				
Factor V Leiden	12 (15)	9 (13.24)	3 (25)	0.3
Prothrombin 20210A mutation	5 (6.25)	4 (5.88)	1 (8.33)	0.75
Deficiency of antithrombin, protein C or S deficiency	5 (6.25)	5 (7.35)	0 (0)	0.62
Antiphospholipid syndrome	5 (6.25)	4 (5.88)	1 (8.33)	0.95
Laboratory investigations				
Creatinine, µmol/L	71 (60-78.25)	70.5 (60–78)	75 (62.5–82.5)	0.33
Glucose, mmol/L	4.84 ± 0.51	4.82 ± 0.51	4.99 ± 0.54	0.28
White blood cells, × 10^9^/L	6.63 ± 1.7	6.72 ± 1.68	6.13 ± 1.79	0.27
Platelets, × 10^9^/L	215.5 (179.5–259)	209 (167.5–259)	227 (197.5-290.5)	0.16
Hemoglobin, g/dL	13.85 ± 1.34	13.89 ± 1.38	13.65 ± 1.13	0.58
INR	1.02 ± 0.09	1.01 ± 0.1	1.03 ± 0.09	0.52
APTT, s	29.2 (28.1–31.2)	29.2 (28.5–31.3)	27.9 (26.6–29.5)	0.03
Fibrinogen, g/L	2.9 (2.57–3.21)	2.87 (2.57–3.18)	3.08 (2.7–3.35)	0.23
C-reactive protein, mg/L	1.85 (0.92–3.2)	1.92 (0.84–3.2)	1.32 (0.98–2.85)	0.54
D-dimer, ng/mL	256(207–324)	252 (210–319)	263(199–346)	0.89
Tissue plasminogen activator, ng/mL	9.56 ± 2.06	9.56 ± 2.18	9.53 ± 1.25	0.97
Plasminogen activator inhibitor-1, ng/mL	24.82 ± 5.68	24.7 ± 5.88	25.46 ± 5.59	0.67
Lipoprotein(a) (mg/dL)^a^	15.3 (9.8–27.7)	14.15 (8.85–25.25)	28.3 (18.9–35.6)	0.001
Fibrin clot properties^a^				
K_s_, 10^−9^ cm^2^	6.57 ± 0.95	6.7 ± 0.86	5.77 ± 1.1	0.001
Lag phase, s	40 (38–43)	40.5 (38–43)	40 (37–43)	0.58
∆Abs (405 nm)	0.89 ± 0.06	0.85 ± 0.06	0.88 ± 0.06	0.4
CLT, min	99.68 ± 17.23	99.1 ± 17.22	102.9 ± 17.66	0.67
D–D_max_, mg/L	4.34 ± 0.51	4.28 ± 0.47	4.72 ± 0.55	0.008
D–D_rate,_ mg/L per minute	0.069 ± 0.004	0.069 ± 0.004	0.067 ± 0.005	0.23

Values are given as mean ± standard deviation or median (interquartile range)

*Patients without recurrence vs patients with recurrence of CVST

^a^P values were adjusted for fibrinogen

A single sinus was affected in 43 (53.75%) patients. The most common site of CVST, which involved single sinus was the transverse sinus in 17 (21.25%) patients, followed by the sagittal sinus in 16 (20%) and the sigmoid sinus in 6 (7.5%) patients. One patient had venous sinus thrombosis isolated to the cavernous sinus. 37 (46.25%) patients had CVST in two or more sinuses.

Median Lp(a) in our cohort was 15.3 (9.8–27.7) mg/dL. The Lp(a) levels were positively associated with fibrinogen (r = 0.33, P = 0.003).No other correlations between Lp(a) levels and age, gender, number of sinuses affected, BMI, routine laboratory investigations, including D-dimer were observed. Moreover, there were no associations of Lp(a) levels with tPA and PAI-1. Patients with CVST in other locations had significantly increased Lp(a) levels compared with the remainder (30.3 [18.7–33.3] versus 14.2 [9.5-25.05] mg/dL, P = 0.016). In patients with the remaining sites affected by CVST compared with the rest no differences in Lp(a) levels were found.

**Fig. 1:**
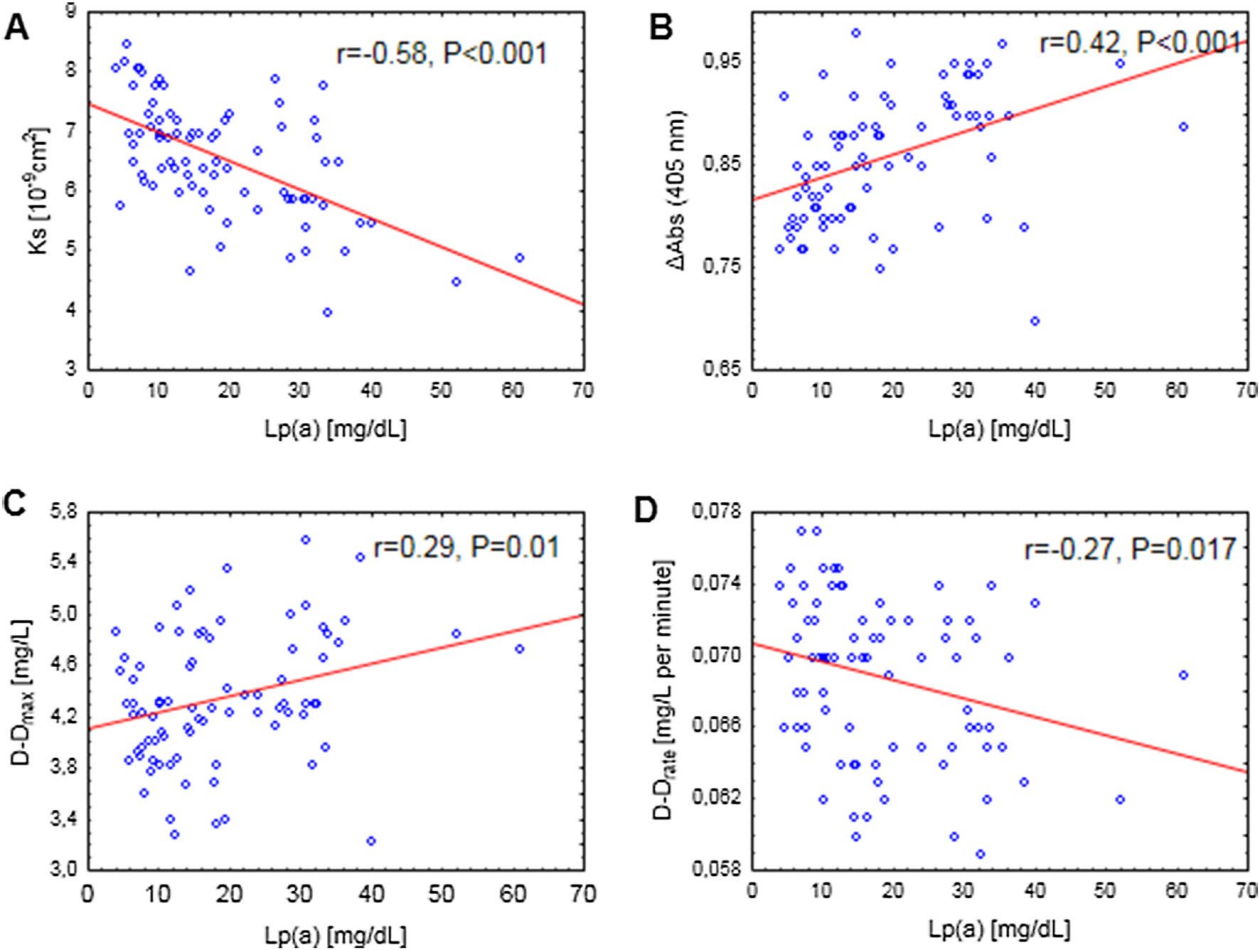
Correlations between Lp(a) and K_s_ (panel A), ∆Abs (405 nm, panel B), D–D_max_ (panel C), D–D_rate_ (panel D)

As shown in Fig. 1, Lp(a) was strongly negatively correlated with K_s_ (r = − 0.58, P < 0.001, panel A) and positively associated with maximum absorbance of fibrin gels (r = 0.42, P < 0.001, panel B), indicating increased plasma clot density. Lag phase on turbidimetric curves was not related to Lp(a). Moreover, Lp(a) showed a positive correlation with D–D_max_ (r = 0.29, P = 0.01, panel C), which indicates increased clot density at high Lp(a) levels. Lp(a) showed an inverse association with D–D_rate_ (r = − 0.27, P = 0.017, panel D), but no association was observed between Lp(a) and CLT.

### Follow-up data

During a median follow-up of 53 months (interquartile range 40–59), recurrence of CVST was observed in 12 patients (15%) with an annual rate of 3.7% (95% CI 1.09–8.77%) person-year. The rate at first year was 1.25%. A median time since anticoagulation withdrawal to the second event was 26 months (19.8–34.3). Provoked recurrences were noted in 5 (42%) patients. Males were overrepresented in the group with recurrence of CVST with no other differences in basic characteristics and routine laboratory investigations including fibrinogen. There were also no differences in thrombophilic risk factors related to CVST recurrences (Table 1). Patients with recurrent CVST had 4.5% shorter APTT compared to the remainder, but still all values were within the reference range. Recurrence of CVST occurred predominantly in patients with multiple sinuses affected at the first event (n = 8, 66.67%). In 8 patients with recurrent CVST the involved sites during the first episode were the sagittal and sigmoid sinuses (66.67%), followed by the transverse sinus (50%).

**Fig. 2:**
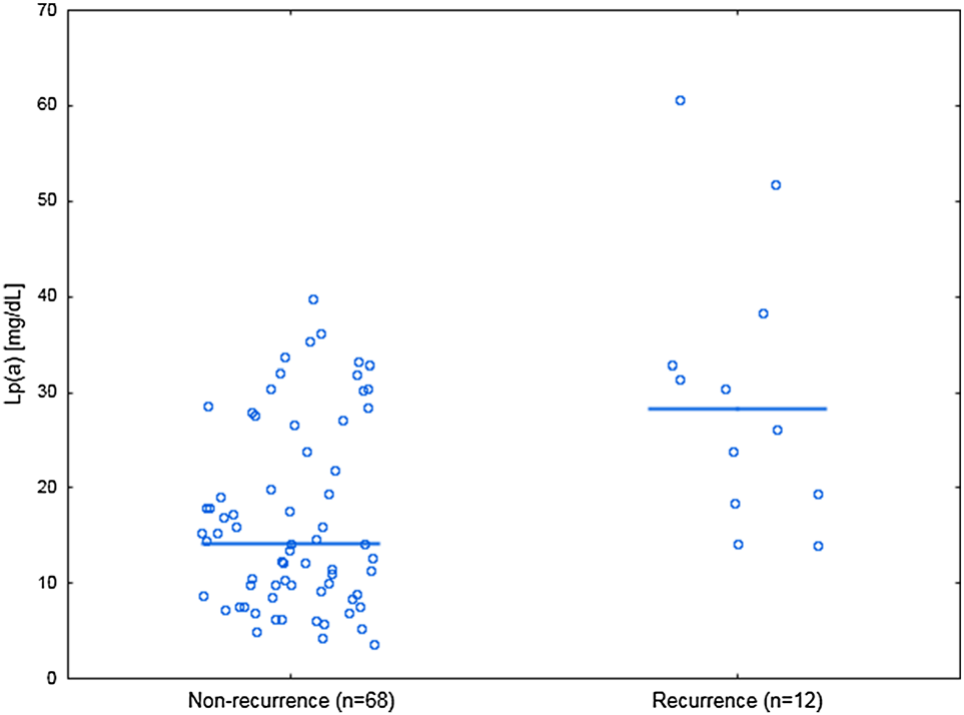
Lp(a) levels for patients with and without recurrence of CVST. Horizontal lines denote the medians for each group

The levels of Lp(a) were 2 times higher in patients with recurrence of CVST compared to the remainder (Table 1; Fig. 2)

**Fig. 3:**
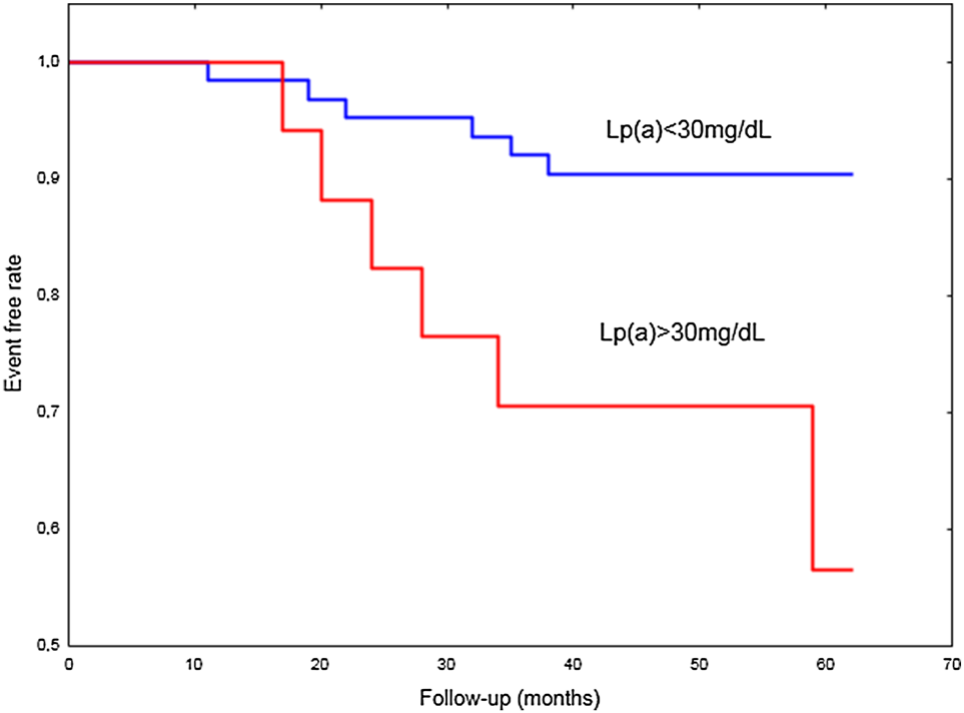
Kaplan–Meier curves of recurrent CVST during follow-up with regard to Lp(a) (log-rank P = 0.01)

The risk of recurrent CVST was 3.9-fold higher among 17 (21.25%) patients with Lp(a) > 30 mg/dL compared with the remainder (HR adjusted for fibrinogen = 3.9, 95% CI 1.23–12.4; Fig. 3).

Recurrence of CVST was associated with 14% lower K_s_ and 10% higher D–D_max_, measured at the time of anticoagulation withdrawal. No other differences between fibrin variables were noted (Table 1).

Multiple logistic regression model adjusted for fibrinogen and sex showed that CVST recurrence was independently associated with higher levels of Lp(a) (odds ratio 1.09, 95% confidence interval 1.02–1.16) (Table 2).

**Table 2:** Multiple logistic regression models adjusted for fibrinogen for patients with recurrence of CVST

	Models with fibrinogen and sex		Multivariable	
	Odds ratio (95%CI)	P	Odds ratio (95%CI)	P
Lipoprotein(a) (mg/dL)	1.11 (1.03–1.17)	0.007	1.09 (1.02–1.16)	0.01
D–D_max_, mg/L	5.72 (1.12–29.29)	0.036	3.84 (0.87–16.93)	0.08
K_s_, 10^−9^cm^2^	0.29 (0.11–0.76)	0.012	0.58 (0.22–1.54)	0.27
∆Abs (405 nm)*	1.49 (0.45–4.9)	0.52		
CLT, min	1.01 (0.97–1.05)	0.75		
Lag phase, s	1.01 (0.85–1.29)	0.68		
D–D_rate,_ mg/L per minute**	0.56 (0.13–2.42)	0.43		

*multiplied by 10

**multiplied by 100

## Discussion

To our knowledge it is the first study to demonstrate that elevated Lp(a) is often observed in patients following CVST and notably, this variable can predict recurrent CVST during a few years of follow-up. This study extends the previous findings in the role of Lp(a), suggesting its contribution to another thrombotic disorder. Importantly, we found that in patients following CVST, elevated Lp(a) levels are associated with decreased fibrin clot permeation, which is consistent with observations reported in other patient populations at risk of thromboembolic events [25–28, 30, 31]. Moreover, a positive correlation between Lp(a) and D–D_max_ was observed in our cohort and patients on long-term haemodialysis, strengthening the association between increased plasma clot density and higher levels of Lp(a) [31]. Importantly, in the current study Lp(a) correlated inversely with D–D_rate_, indicating impaired fibrinolysis, which is in line with previous findings [28]. No such association was found between CLT and Lp(a). This study suggests that higher levels of Lp(a) may contribute to prothrombotic fibrin clot phenotype in patients with CVST. However, a novel finding is identification of Lp(a) as an independent risk factor for CVST recurrences, which suggests additional mechanisms linking Lp(a) with CVST beyond fibrin-related effects.

Impaired fibrinolysis in patients with higher levels of Lp(a) could be explained partly by inhibition of plasminogen binding to fibrin and the interference with conversion of Glu-plasminogen to Lys-plasminogen and therefore reduction of the amount of plasmin in the tissue-type plasminogen activation [16, 17, 32]. Recently, Stachowicz et al. found in plasma fibrin clots from 4 patients with venous thromboembolism the presence of apo(a), which confirms that Lp(a) binds to clots and might affect clot structure and function including susceptibility to lysis [33]. The associations of Lp(a) with clot properties reported previously and the current work appear to confirm this hypothesis.

Regarding recurrences during follow-up, we confirmed previous observations made in a group of 50 patients indicating that K_s_ and D–D_max_ are factors determining CVST [7]. Males were more likely to experience recurrence of CVST in the present study, which is consistent with findings of Martinelli et al. [6]. The current study shows that impact of elevated Lp(a) on recurrent CVST is observed also after adjustment for fibrinogen and sex.

Our study has several limitations. Firstly, the size of the study group was limited particularly in the subgroup analysis. Secondly, each analyzed variable was assessed only once at a single time and thus analyses should be interpreted with caution. Moreover, the results of this study cannot be easily extrapolated to patients older than 60 years and individuals with previous thromboembolic events. We did not analyze genetic polymorphisms of Lp(a) gene and its effects on association between Lp(a) levels and fibrin clot properties. However, our previous work showed that in apparently healthy individuals and patients following myocardial infarction the number of kringle IV repeats and pentanucleotide repeats were positively correlated with K_s_ and negatively with clot lysis time, t_50%_ [30]. Thus, it can be assumed that elevated Lp(a) levels are associated with unfavorable clot properties.

In conclusion, we reported for the first time that increased Lp(a) characterizes subjects with higher risk of CVST recurrence after cessation of anticoagulation, which could be in part driven by prothrombotic fibrin clot phenotype expressed by denser clot structure and impaired fibrinolysis. A value of elevated Lp(a) as a risk factor of CVST remains to be assessed in other populations. The present study has potential practical implications. It might be speculated that patients with Lp(a) > 30 mg/dl should be anticoagulated on the long-term basis given elevated risk of CVST recurrences. However, in view of bleeding risk, such approach should be tested in a larger study.
